# 3D Printing of CNT- and YSZ-Added Dental Resin-Based Composites by Digital Light Processing and Their Mechanical Properties

**DOI:** 10.3390/ma16051873

**Published:** 2023-02-24

**Authors:** Minhyuk Son, Kati Raju, Jaemin Lee, Jinsik Jung, Seik Jeong, Ji-in Kim, Jaehun Cho

**Affiliations:** School of Advanced Materials Science and Engineering, Kumoh National Institute of Technology, Gumi 39177, Gyeongbuk, Republic of Korea

**Keywords:** 3D printing, dental resin-based composites, digital light processing, CNT, YSZ

## Abstract

This study demonstrates the successful 3D printing of dental resin-based composites (DRCs) containing ceramic particles using the digital light processing (DLP) technique. The mechanical properties and oral rinsing stability of the printed composites were evaluated. DRCs have been extensively studied for restorative and prosthetic dentistry due to their clinical performance and aesthetic quality. They are often subjected to periodic environmental stress, and thus can easily undergo undesirable premature failure. Here, we investigated the effects of two different high-strength and biocompatible ceramic additives, carbon nanotube (CNT) and yttria-stabilized zirconia (YSZ), on the mechanical properties and oral rinsing stabilities of DRCs. Dental resin matrices containing different wt.% of CNT or YSZ were printed using the DLP technique after analyzing the rheological behavior of slurries. Mechanical properties such as Rockwell hardness and flexural strength, as well as the oral rinsing stability of the 3D-printed composites, were systematically investigated. The results indicated that a DRC with 0.5 wt.% YSZ exhibits the highest hardness of 19.8 ± 0.6 HRB and a flexural strength flexural strength of 50.6 ± 6 MPa, as well as reasonable oral rinsing steadiness. This study provides a fundamental perspective for designing advanced dental materials containing biocompatible ceramic particles.

## 1. Introduction

Digital light processing (DLP) is one of the emerging and fascinating 3D printing technologies which can be used for the fabrication of desired complex-shaped materials through the photopolymerization of photocurable resins. DLP is a simple, versatile, and time-saving technique, and its features include low cost and environmental sustainability. It has been used to produce a variety of materials including not only photosensitive resins but also composites containing ceramics, biomaterials, and metals [1,2,3,4,5,6]. Recent studies have successfully demonstrated the potential of DLP technology in the 3D printing of ceramic-loaded slurries for different works in the medical research field, including biomaterials, bone scaffolds, and dental implants [7,8,9,10,11]. More recently, the effect of the post-curing time on the color stability of tooth-colored 3D resins [9] and the wear volume of interim crowns fabricated using DLP was reported [11]. Before printing, the desired 3D shapes can be designed and confirmed through suitable computer design software, which is one of the beneficial and crucial steps involved in this technology.

Dental resin-based composites (DRCs) have been extensively studied for restorative and prosthetic dentistry due to their suitable functional properties, adequate mechanical properties, sufficient clinical performance, and aesthetic quality [12,13,14,15,16,17,18,19,20,21,22,23,24]. Recent research developments in DRCs and unique approaches to enhance various properties can be found elsewhere in the literature [19,20,22]. DRCs can easily experience undesirable premature failure because they are often subjected to periodic environmental stresses. Hence, in designing and developing new DRCs, especially for dental applications, a fundamental understanding of the mechanical behavior of DRCs is necessary together with that of biocompatibility. Many studies are devoted to the improvement of the mechanical properties of DRCs by incorporating different materials [15,21,22,23,24,25,26,27,28,29,30,31,32,33,34]. For instance, Xu et al. [25] and Yan et al. [34] investigated the effects of nanosized calcium phosphate and zirconia additives on the mechanical properties of dental composites, respectively.

Carbon nanotubes (CNTs) are considered promising materials in various research fields, including aerospace, automobile, textile, and dentistry, due to their outstanding mechanical properties [35,36]. CNTs were added to improve the electrical, mechanical, and elastic properties of numerous polymer/CNT nanocomposites processed using different techniques [37,38,39,40,41,42,43,44,45,46]. For example, the addition of 1 wt.% CNT nanoparticles enhanced the elastic modulus of high-density polyethylene composites by 24% [40]. On the other hand, yttria-stabilized zirconia (YSZ) is one of the best ceramic materials that is used for dentistry and prostheses applications due to its excellent biocompatibility [47,48]. Furthermore, YSZ possesses both remarkable strength and fracture toughness, which is usually mutually exclusive for most of the materials. Moreover, results showed that the addition of YSZ particles enhanced the thermomechanical, physical, and biological properties of some ceramic- and polymer-based composites [49,50,51]. Nevertheless, studies on 3D-printed DRCs with CNT or YSZ additives are seldom found in the literature. Given the above facts, this study aims to fabricate 3D DRCs using the DLP technique and to evaluate their mechanical properties and oral rinsing stabilities. Microstructure analyses of the 3D DRCs with various wt.% of ceramic additives are performed, and potential fracture mechanisms are suggested.

## 2. Experimental Procedure

In this study, commercially available polyacrylate-based photocurable resin (3D-Polymer, Shade-A2, Myungmun Dental Corp., Daegu, Korea) was used. It contains photosensitive resins of bisphenol A diacrylate and tri (propylene glycol) diacrylate, a photoinitiator of diphenyl (2,4,6-trimethyl benzoyl) phosphine oxide, and aesthetic additives of TiO_2_ and Sicotan^®^ Yellow as ingredients. Carbon nanotubes (CNT Regular, Carbon Nano Tech., Pohang, Korea) and yttria-stabilized tetragonal zirconia (YSZ, TZ-3Y-E grade granules, Tosoh Corporation, Tokyo, Japan) were used as ceramic additives to prepare various DRCs. Then, various amounts of CNTs or YSZ in wt.% were added to the pure resin and were thoroughly stirred to ensure a homogeneous distribution. Considering the high-viscosity nature of pure resin, stirring was performed rigorously at 1000 rpm for 2 h at 100 °C by adding 1 wt.% of dispersant (DISPERBYK-111, BYK-Chemie GmbH, Abelstraße, Germany). After thorough mixing of CNT or YSZ with basic resin, the rheological behavior of the slurries was measured using a computer-controlled rheometer (RM200 CP4000 PLUS, Lamy Rheology, Champagne au Montd’Or, France) at room temperature. The average particle size (*d_50_*) and particle size distribution of as-received raw materials were measured using a laser-diffraction particle analyzer (PSA, Mastersizer 2000, Malvern Panalytical, Malvern, UK).

After designing the desired 3D structures with computer-aided design software, specimens were manufactured using a DLP 3D printer (IMC-96, Carima Co., Ltd., Daejeon, Korea), and cleaning was performed using isopropyl alcohol to remove uncured extra resin on surfaces. All the printing parameters used in the present work are provided in Table 1.

**Figure 1:**
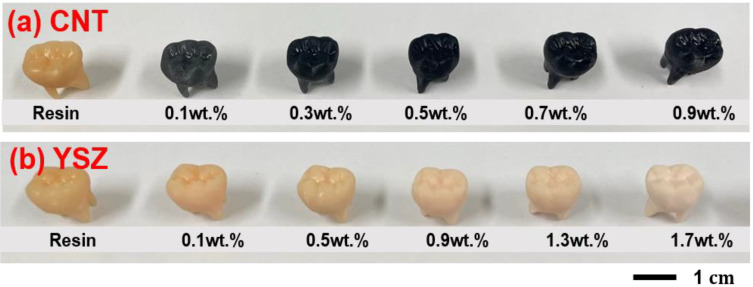
Digital camera images of various (**a**) CNT- and (**b**) YSZ-added 3D-printed teeth with dimensions of 10 × 10 × 10 mm^3^. With increasing CNT and YSZ contents, the teeth become darker and brighter, respectively.

All specimens were additionally cured using a post-UV curing system (CL300Pro, Carima, Co., Ltd., Korea). Subsequently, vacuum drying was performed at 60 °C for 5 h after ultrasonic cleaning. Figure 1 shows typical digital camera images of various CNT- and YSZ-added 3D-printed teeth.

To compare the strength of the printed specimens, a three-point bending test was performed with a universal testing machine (Quasar 100, Galdabini, Cardano al Campo, Italy). For this purpose, bar specimens with dimensions of 9*^w^* × 3*^t^* × 60*^l^* mm^3^ were produced using 3D printing, and an average of at least five measurements was reported here. An HRB-type Rockwell indenter (DTR-200N, DTNT, Incheon, Korea) with a 1/16-inch diamond tip was used to measure the hardness. Next, 3D-printed specimens were subjected to a load of 100 kg·f. Hardness was measured at three different locations (top, side, and bottom sides). The hardness reported here is an average of at least ten measurements. The oral rinsing stabilities of the 3D-printed specimens with different additive amounts were evaluated. For this purpose, the specimens were immersed in commercially available Listerine^®^ oral solution for 3 days. The changes in height, width, and weight of the specimens were measured after complete drying at 60 °C for 5 h in a vacuum dryer. Finally, the microstructure of the fractured surfaces after flexural strength measurements was examined using scanning electron microscopy (SEM, JSM-6500F, JEOL, Tokyo, Japan).

## 3. Results and Discussion

**Figure 2:**
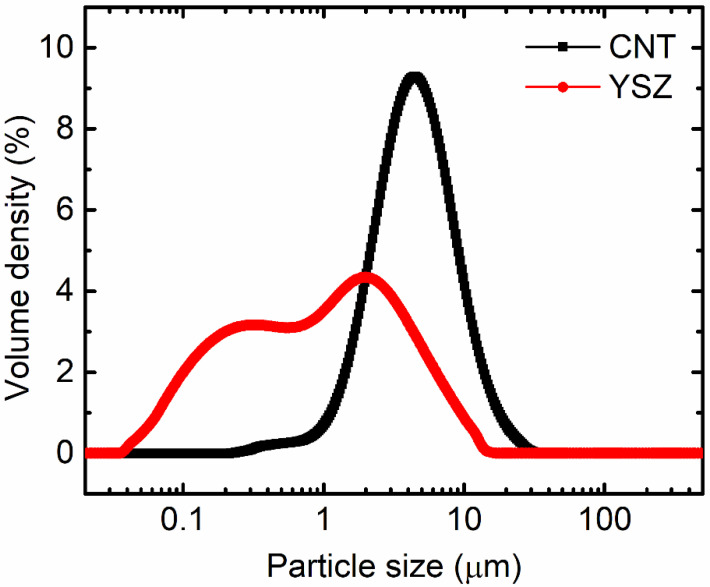
PSA analysis of as-received CNT and YSZ raw materials. Average particle size of CNT is 4.4 μm, whereas that of YSZ is 1.0 μm.

PSA analysis of as-received CNT and YSZ raw materials was performed before preparing slurries to understand the effect of average particle sizes and particle size distributions on the properties of DRCs. Relatively smaller particle sizes are required to enhance the homogeneous distribution of particles and the mechanical properties of the composites. PSA analysis (Figure 2) revealed that the average particle size, *d_50_*, was about 4.4 μm for CNT due to severe agglomeration, whereas it was only 1.0 μm for YSZ powders. Moreover, *d_10_* and *d_90_* were 1.9 and 10.1 μm, respectively, for CNT. Relatively lower values of 0.1 and 4.7 μm for d*_10_* and *d_90_*, respectively, were found for YSZ. Moreover, the particle size distribution curve for YSZ was relatively broader than CNT.

**Figure 3:**
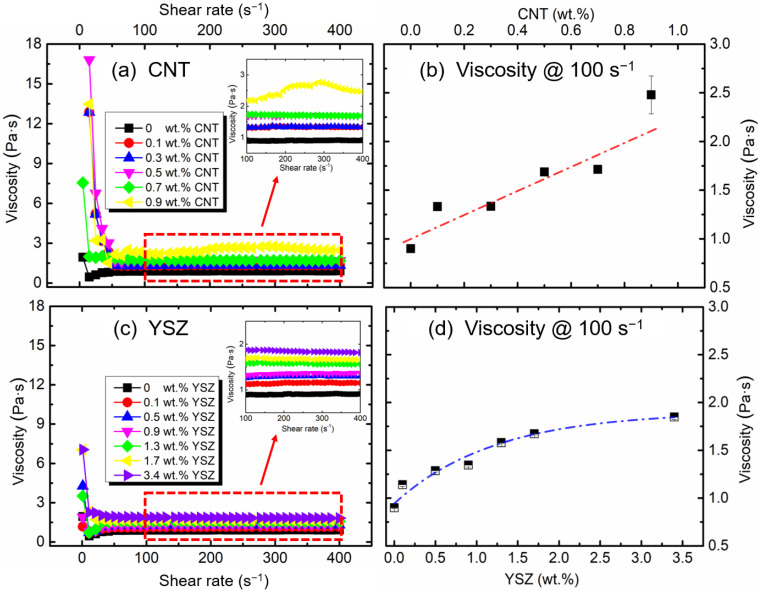
Rheological behaviors of slurries containing (**a**) CNT and (**c**) YSZ at various wt.% of the additives as a function of shear rate at room temperature. Comparison of viscosity of (**b**) CNT− and (**d**) YSZ−bearing resins at a constant shear rate of 100 s^−1^.

The rheological behavior of the CNT and YSZ bearing resins was also studied before 3D printing by measuring the viscosity as a function of the shear rates at room temperature. Figure 3 compares the viscosity of slurries containing CNT and YSZ particles at various wt.% concentrations. All suspensions display a shear-thinning behavior. For easy understanding, the viscosity data were compared at the same scale at a particular shear rate of 100 s^−1^ in Figure 3b,d, respectively, for CNT and YSZ additions. Viscosities are found to increase with increasing amounts of both CNT and YSZ additions. The viscosity of pure resin was 0.9 Pa·s at 100 s^−1^. The viscosity of CNT-added samples can quickly reach up to 2.5 Pa·s at 100 s^−1^. On the other hand, YSZ-bearing slurries reach the viscosity of 1.8 Pa·s at a shear rate of 100 s^−1^ with 3.4 wt.% addition of YSZ. These results indicate that the viscosity of YSZ-added slurries is much lower than CNT-added samples; hence, it was printed up to 3.4 wt.% of YSZ without hindrance under a light intensity of 2.8 mW/cm^2^. However, for CNT-added slurries, it was unprintable beyond the 0.9 wt.% addition of CNT due to high viscosity.

**Figure 4:**
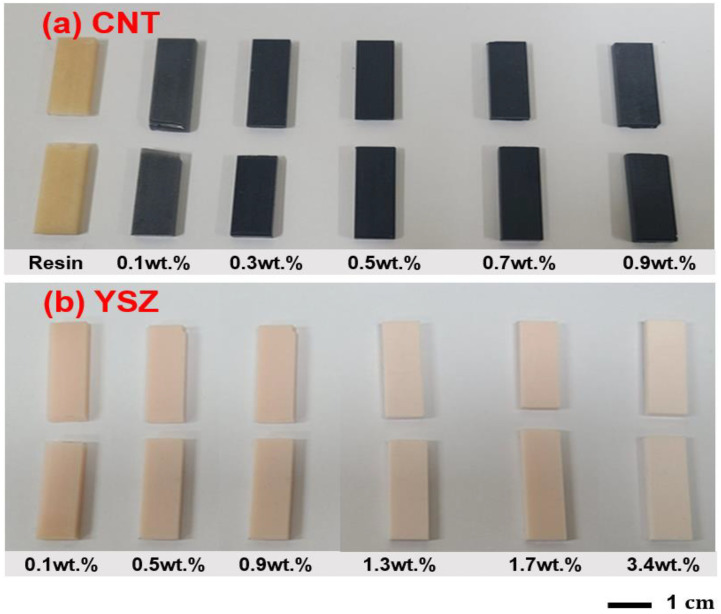
Digital camera images of various (**a**) CNT- and (**b**) YSZ-added bar specimens after three-point bending test.

**Figure 5:**
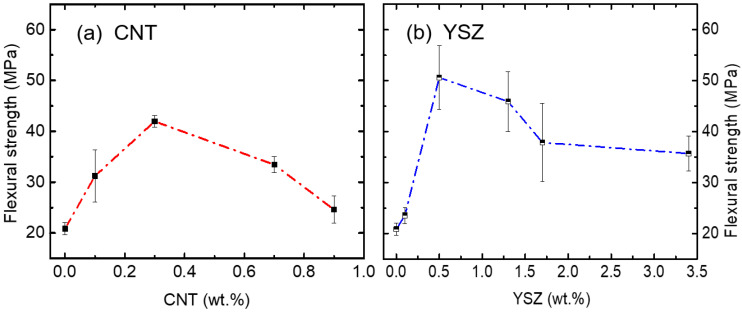
Flexural strength of (**a**) CNT- and (**b**) YSZ-added specimens as a function of additive amounts. The flexural strength increases up to 41.9 ± 1 MPa when 0.3 wt.% of CNT is added and decreases significantly thereafter. Whereas the 0.5 wt.% YSZ-added specimen shows a maximum flexural strength of 50.6 ± 6 MPa and maintains its strength higher than a value of 35.7 ± 3 MPa after increasing YSZ content to 3.4 wt.%.

The mechanical strength of the composites was evaluated by measuring the three-point flexural bending test. Digital camera images of various CNT- and YSZ-added specimens after fracture are presented in Figure 4. Flexural strength data were compared in Figure 5 at the same scale for both CNT- and YSZ-added specimens. The strength of pure resin was 20.8 ± 1 MPa. It can be observed from the figure that the addition of CNT increases the strength to the maximum of 41.9 ± 1 MPa for 0.3 wt.% addition, and it decreases thereafter with further increasing CNT content. The strength reaches a minimum of 24.6 ± 2 MPa with the addition of 0.9 wt.% CNT. On the other hand, in the case of YSZ-bearing specimens, the maximum strength of 50.6 ± 6 MPa was achieved through the addition of 0.5 wt.% YSZ. Further, it decreases to the minimum of 35.7 ± 3 MPa with increasing YSZ content to 3.4 wt.%. These flexural strength values are comparable with the reported other DRCs (27~80 MPa) in the literature [24]. Moreover, both alumina- and YSZ-added (0.5 wt.% each) polylactic-based composites displayed a flexural strength of 53.19 MPa [50]. From these results, it can be concluded that only certain amounts of CNT and YSZ are sufficient to achieve maximum strength. It is worth noting that a subtle decrease in flexural strength was observed for the 3.4 wt.% YSZ addition, in drastic contrast to the CNT case.

To evaluate the strength essentially for dental applications, Rockwell hardness was measured mainly at three locations (top, side, and bottom sides) of the 3D-printed teeth.

**Figure 6:**
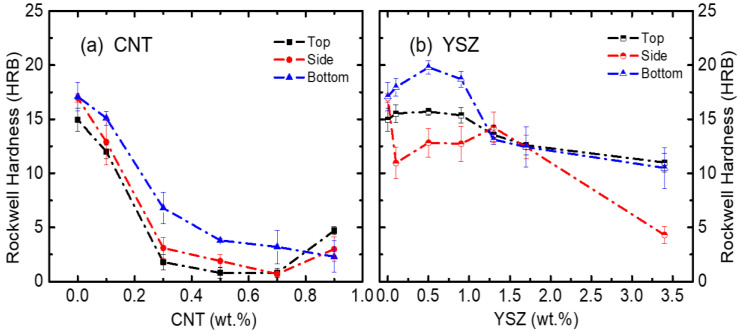
Variation of Rockwell hardness with increasing contents of (**a**) CNT and (**b**) YSZ particles, respectively, at top, side, and bottom regions of 3D-printed teeth. Rockwell hardness decreases with increasing CNT content, whereas it shows hardness enhancement for YSZ-added specimens, especially at the top and bottom regions when YSZ content is lower than 0.9 wt.%.

**Figure 7:**
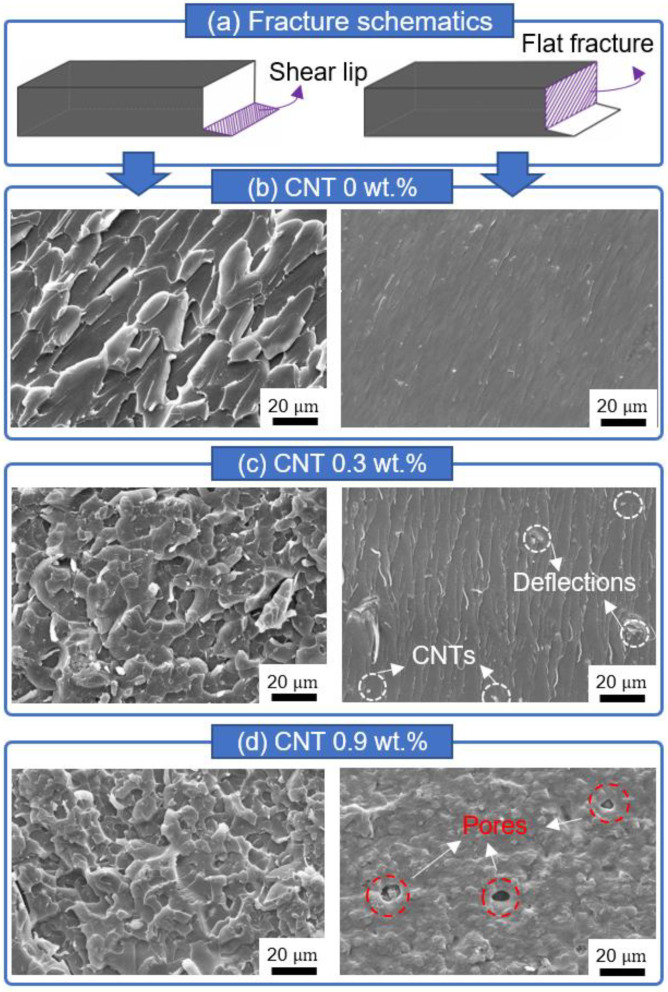
Typical SEM images of CNT-added specimens after the three-point bending test: (**a**) Schematic description shows typical fracture surfaces of combined shear lip and flat fracture. (**b**) SEM images of pure resin show coarse dimples with torn-off traces on the shear lip region, whereas it exhibits a smooth and even surface on the flat fracture region. (**c**) Fracture surface of the 0.3 wt.% CNT-added specimen contains smaller dimples decorated with CNT particles, deflecting crack propagation. (**d**) Fracture surface of the 0.9 wt.% CNT-added specimen exhibits a large number of pores detrimental to mechanical strength.

**Figure 8:**
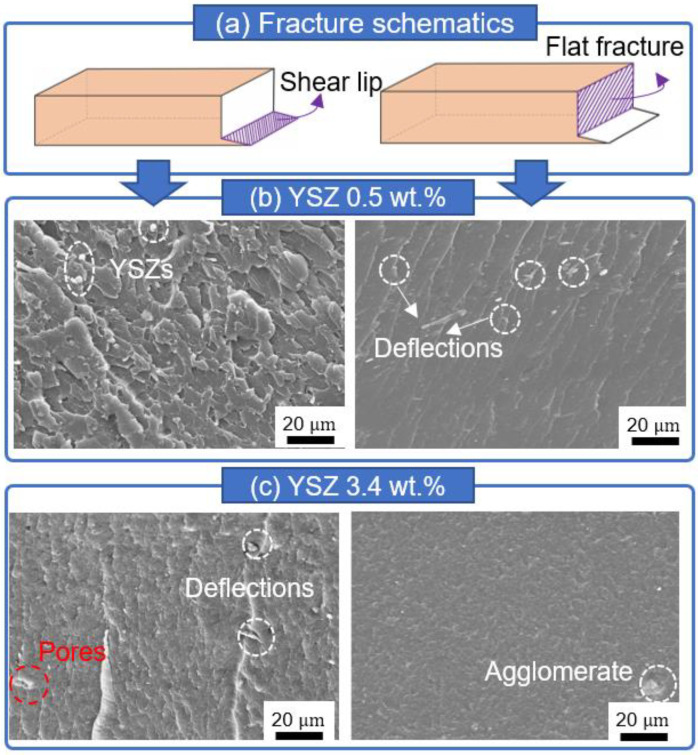
Representative SEM images of YSZ-added specimens after the three-point bending test: (**a**) Schematic illustration shows that a typical fracture mode of YSZ-added specimens consists of both shear lip and flat fracture. (**b**) Fracture surface of the 0.5 wt.% YSZ-added specimen contains smaller dimples than that of pure resin and white YSZ particles, deflecting crack propagation. (**c**) SEM images of the 3.4 wt.% YSZ specimen shows pores and agglomerates harmful to mechanical strength.

Rockwell hardness data of both the CNT- and YSZ-added samples were compared at the same scale in Figure 6. The hardness values are comparable for all three locations, and this demonstrates that the 3D-printed teeth possess uniform strength in all directions. The hardness of the CNT-added samples is much lower than the YSZ-added specimens for all wt.% concentrations. The hardness continuously decreases with increasing CNT content, whereas it remains almost constant in the case of the YSZ samples at the top location. The hardness decreases drastically from 16 HRB to ~4 HRB after adding CNT additives at all three locations. On the other hand, significant hardness enhancement can be achieved through the addition of 0.5 wt.% of YSZ, especially at the bottom side. To understand the variation of the mechanical properties of the composites with changing wt.% concentrations of CNT and YSZ particles, an SEM study was performed on the fracture surfaces after the three-point bending test. The microstructure after fracture was compared in Figure 7 and Figure 8, respectively, for CNT- and YSZ-added specimens. SEM observations revealed that both shear lip and flat fracture mechanisms are involved in the fracture mode. A schematic illustration of the shear lip and flat fracture mechanisms is shown in Figure 7a. SEM images of pure resin are presented in Figure 7b. On the shear lip of the specimen, coarse dimples with torn-off traces can be observed, and, through this, the direction of the crack propagation can be known. In a flat fracture, the entire surface is smooth and even. This indicates that there exists little resistance to the progress of cracks in the flat fracture region. Figure 7c shows the SEM images of the specimen prepared by adding 0.3 wt.% of CNT to the pure resin. On the shear lip of the specimen, the sizes of the dimples are much smaller, and the CNT particles are evenly distributed throughout the specimen. Moreover, river patterns can be observed in flat fracture regions (Figure 7c). The CNT particles are located in the middle of the crack trace through the area marked with white circles, and, thus, the 0.3 wt.% CNT-added specimen has a relatively high resistance to crack propagation, as evidenced by the highest flexural strength of 41.9 MPa. Figure 7d shows the SEM images of the specimen prepared by adding 0.9 wt.% of CNT to the pure resin. There exists a higher density of dimples on the shear lip region; however, a large number of pores are present on the flat surface area. The appearance of pores on the samples may be attributed to the addition of CNTs, as increasing the amount of CNTs in the resin mixture can increase its local viscosity. This increased viscosity can hinder the complete filling of the free space left by the base plate, leading to the formation of voids in the printed structure. The porosity of the samples can also be influenced by the printing process parameters, such as layer thickness, exposure time, and resin flow rate. However, further studies are needed to fully understand the mechanisms behind pore formation with different CNT amounts.

Typical SEM images of 0.5 and 3.4 wt.% added YSZ are shown in Figure 8b,c, respectively. More dimples can be observed on the shear lip region of the 0.5 wt.% YSZ specimen. Figure 8b shows that YSZ particles are evenly distributed. In the area where the flat fracture occurred, the river pattern can be confirmed as being similar to the CNT counterparts. However, the crack traces were deflected by the presence of YSZ particles, and thus this specimen has higher hardness and flexural strength than those of the pure resin specimen. Figure 8c presents the SEM images of the 3.4 wt.% YSZ specimen and reveals the existence of many dimples along with the presence of pores on the shear lip region. When the YSZ content is increased from 0.5 to 3.4 wt.%, crack deflection can be observed more often due to the increased number of YSZ particles. However, due to the presence of pores acting as a stress concentrator, the flexural strength of the 3.4 wt.% YSZ-added specimen is lower than that of its 0.5 wt.% YSZ-added counterpart. Agglomeration of YSZ particles can also be observed in this specimen. This is due to the excessive addition of YSZ particles, leading to an increase in the local viscosity of the mixed resin.

**Figure 9:**
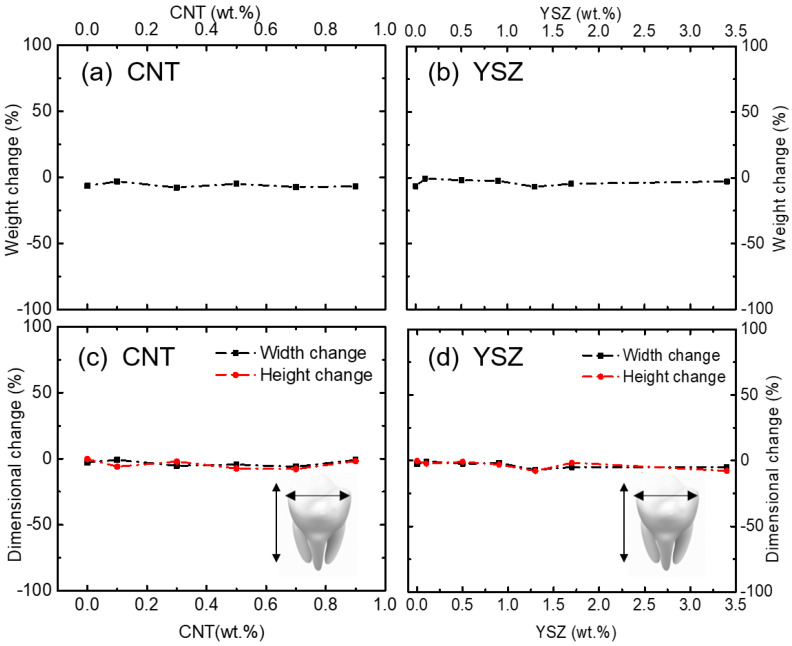
(**a**,**b**) Weight and (**c**,**d**) dimensional changes of 3D-printed teeth for CNT− and YSZ−added specimens, respectively, after 3 days of treatment with Listerine^®^. No significant changes in weight and size were observed for both CNT− and YSZ−added specimens.

During the surgical treatment process, dental restorative materials are often used along with oral mouth rinses. To assess the stability of the prepared teeth in the present work, preliminary studies were performed using Listerine^®^ solution for 3 days. Listerine^®^ is one of the most popular mouthwashes used to maintain hygienic teeth by protecting them from several harmful microorganisms [52,53,54,55,56,57,58]. Listerine^®^ is composed of several essential oils and some other organic compounds. Therefore, it is reasonable to study the effect of Listerine^®^ on these prepared samples and the results are compared in Figure 9 for CNT- and YSZ-containing composites. It is clear from the data that the weight of both the CNT- and YSZ-added samples remain almost unchanged and exhibit excellent stabilities. The dimensional changes after 3 days of immersion were less than 0.8 mm for both CNT and YSZ specimens irrespective of the wt.% of additives.

## 4. Conclusions

In conclusion, 3D DRCs containing biocompatible CNT and YSZ particles were successfully fabricated through the DLP technique. Various dental resin matrices containing different wt.% of CNT or YSZ were printed after examining the rheological behavior of slurries. Mechanical properties such as Rockwell hardness and the flexural strength of the 3D-printed composites were systematically investigated. Moreover, oral rinsing stabilities were evaluated using a Listerine^®^ solution. The results indicated that a DRC with a 0.5 wt.% YSZ addition exhibits the highest hardness and flexural strength of 50.6 ± 6 MPa, as well as reasonable oral rinsing stability. This study provides a fundamental perspective for designing advanced dental materials containing ceramic particles. The findings of the study could potentially contribute to the development of more effective and efficient DRCs, leading to better clinical outcomes and paving the way for future advancements in 3D printing technology in dentistry and beyond.

## Figures and Tables

**Table 1:** All the DLP printing parameters used in the present work.

UV wavelength of DLP printer:	405 nm
Light intensity:	2.8 mW/cm^2^
Curing time for each layer:	7 s
Layer thickness:	50 μm
Curing time after printing:	10 min
Resin temperature while printing:	25 °C

## Data Availability

The experimental data will be shared upon a reasonable request to the corresponding author.
